# Health related quality of life in juvenile SLE patients– use of the PedsQL 3.0 rheumatology module

**DOI:** 10.1186/1546-0096-9-S1-P243

**Published:** 2011-09-14

**Authors:** Helena Sousa, Margarida Guedes

**Affiliations:** 1 Santo António-Porto Hospital Center, PORTO, Portugal

## Background

Juvenile SLE (jSLE) is a multisystemic chronic condition associated with significant morbidity consequential to the disease and treatment with negative impact in the quality of life of the patients and their families.

## Aim

to assess the health related quality of life (HRQOL) in the jSLE patients followed in the Pediatric Department of Santo António Hospital - Porto Hospital Center (Portugal).

## Methods

The PedsQL 3.0 Rheumatology Module to adolescents (ages 13-18) (Portuguese translation), including teen self report and parent proxy report, and its instructions was sent by mail to the six jSLE patients of our Pediatric Department. Adolescents and their parents should fill the forms separately and return by mail. The queries were unidentified so the answers were anonymous. Data were analysed in EXCELL.

## Results

All the six queries were receipt with no missing data. Clinical data of the 6 jSLE patients: 5♀/1♂; mean age 15,5±2,07 years, disease onset at 12 (±3,52) years old with mean disease duration 3,42 (±2,4) years. Lupic nephritis in 2 and secondary antiphospholipid syndrome in 3. Mean SLEDAI score 3,3 (maximum 8). All patients were on chloroquine and prednisone. Other immnossupressive drugs included: rituximab; azathioprine and cyclophosphamide courses; mycophenolate mofetil; methotrexate and intravenous immunoglobulins. Antiaggregant therapy in three and ACE-I in 2.

Table 1 presents the results obtained in the teen self report and parent proxy report on the five domains of the PedsQL 3.0 Rheumatology module.

**Table 1:** 

	PedsQL 3.0 Rheumatology module to adolescents (ages 13-18)				
		Teen report		Parent report for teens	

Scale	N° items	Mean (%)	SD	Mean (%)	SD

Pain and hurt	4	66,67	16,14	64,58	20,02

Daily activities	5	91,07	11,23	86,67	17,51

Treatment	7	83,33	10,26	76,77	5,88

Worry	3	**30,56**	38,61	**13,89**	11,38

Communication	3	73,61	23,81	63,89	34,82

## Conclusion

The results were similar in the teen and parent report. In both reports four of the five domains obtained positive (>50%) results. The low results attained in the worry scale alert to the need of improving the psychological support to these patients and their families.

The authors consider important in the approach of jSLE patients the regular evaluation of the HRQOL; besides allowing a more active participation of the adolescent in the disease, gives to the clinician a more holistic information and alarm for underestimated needs.

